# Study on the Structure, Function, and Interface Characteristics of Soybean Protein Isolate by Industrial Phosphorylation

**DOI:** 10.3390/foods12051108

**Published:** 2023-03-05

**Authors:** Yanan Guo, Caihua Liu, Yitong Ma, Lulu Shen, Qi Gong, Zhaodong Hu, Zhongjiang Wang, Xin Liu, Zengwang Guo, Linyi Zhou

**Affiliations:** 1 College of Food Science, Northeast Agricultural University, Harbin 150030, China; 2 College of Food and Health, Beijing Technology and Business University, Beijing 100048, China

**Keywords:** phosphorylation, soybean protein isolate, structure, functional characteristics, interfacial activity

## Abstract

The impacts of industrial phosphorylation on the structural changes, microstructure, functional, and rheological features of soybean protein isolate (SPI) were spotlighted. The findings implied that the spatial structure and functional features of the SPI changed significantly after treatment with the two phosphates. Sodium hexametaphosphate (SHMP) promoted aggregation of SPI with a larger particle size; sodium tripolyphosphate (STP) modified SPI with smaller particle size. SDS–polyacrylamide gel electrophoresis (SDS-PAGE) results showed insignificant alterations in the structure of SPI subunits. Fourier transform infrared (FTIR) and endogenous fluorescence noted a decline in α-helix quantity, an amplification in β-fold quantity, and an increase in protein stretching and disorder, indicating that phosphorylation treatment fluctuated the spatial structure of the SPI. Functional characterization studies showed that the solubility and emulsion properties of the SPI increased to varying degrees after phosphorylation, with a maximum solubility of 94.64% for SHMP-SPI and 97.09% for STP-SPI. Emulsifying activity index (EAI) and emulsifying steadiness index (ESI) results for STP-SPI were better than those for SHMP-SPI. Rheological results showed that the modulus of G’ and G″ increased and the emulsion exhibited significant elastic behavior. This affords a theoretical core for expanding the industrial production applications of soybean isolates in the food and various industries.

## 1. Introduction

Food nutrition and safety are becoming a major concern as a result of the world population’s fast expansion [1]. A key dietary element in people’s everyday lives is soy protein, one of the sources of high-quality plant protein. It is employed in many different food items with rich nutritional content and potent functional qualities [2]. Soybeans have a higher protein level than both cereals and potatoes, and they are filled with all the important amino acids that the human body needs. It can be utilized as a replacement for animal proteins in the food sector since it has nutritional and biological benefits similar to those of animal proteins [3]. However, natural soy protein isolate contains a large number of hydrophobic groups, which seriously affects the solubility and emulsification of soy protein, resulting in its functional properties not being able to be well developed, limiting the application of soy protein in industry [4].

Phosphorylation has been demonstrated to be an effective assay for enhancing the functional features of food proteins [5]. Besides the structural and functional features, the biological roles of food proteins can also be conferred or enhanced by phosphorylation methods [6]. Inorganic phosphates have a long history of being used safely in food, which provides more evidence for their safety. Phosphoryl chloride, phosphorus pentoxide, cyclic phosphoric acid trona, phosphoric acid and trichloro acetonitrile (as a coupling agent), monophenyl phosphonic acid di-chloro compound, sodium tripolyphosphate, and sodium hexametaphosphate are among the chemicals frequently used in food protein phosphate. At present levels, which result in intakes substantially below the acceptable daily intake (ADI) of 70 mg^−1^ kg^−1^ d^−1^, adding inorganic phosphates directly to food is unlikely to have a negative impact on people [7].

Studies on the phosphorylation modification of dietary proteins have been conducted recently. According to Walz et al. [8], adding the right proportions of sodium hexametaphosphate (SHMP) to the sausage batter can prevent efflorescence from growing on dry fermented sausages. According to research by Matheis [9], phosphorylation with POCI_3_ (in the presence and absence of important amino acids) may be a useful technique for enhancing the nutritional value and functional qualities of dietary proteins. According to research by Cen et al. [10], phosphorylation modification of fish gelatin protein (GLP) considerably improved gel strength, textural characteristics, emulsification, and emulsification stability. GLP’s phosphorylation with ultrasound assistance hastened the transition from helix to sheet and random coil. Hu et al. [11] investigated that the use of ultrasound-assisted phosphorylation increased the surface hydrophobicity, protein structure, and lowered protein aggregation to enhance the emulsifying characteristics of goose liver protein. Hu et al. [12] made it possible for us to comprehend the relationship between myofibrillar protein and sodium tripolyphosphate (STPP), tetrasodium pyrophosphate (TSPP), SHMP, and morphology in greater detail. They also showed that phosphates significantly increased gel complexity and decreased average roughness, resulting in a more well-organized and well-built protein gel. 

However, the studies of the above scientists are based on a laboratory basis and the laboratory results and industrial production cannot be reproduced with great variability [13,14], which is not a good guide to the application of phosphate modification in industrial production. This is because phosphorylation modification of SPI in the laboratory is performed by adding phosphate to the rehydrated SPI. Industrial phosphorylation modification of SPI, on the other hand, is performed by adding phosphate directly in the neutralization modification tank during the production of SPI [15]. Therefore, in this study, based on the traditional industrial extraction of SPI, we add phosphate to the neutralization modification tank to phosphorylate SPI to simulate the industrial production of phosphorylated modified soybean protein. Additionally, we study the effect of industrial phosphorylation modification on the structural, functional, and interfacial properties of SPI, and this study provides guidance for the industrial production process of phosphorylated modified soybean isolate.

## 2. Materials and Methods

### 2.1. Materials 

The Yuwang Group (Dezhou, China) supplied soy flakes without fat (52.27% crude protein concentration). From Beijing Dingguo Changsheng Biotechnology Co. Ltd., chemicals such as 2,2′-dithiobis(5-nitropyridine) (DTNP) and 8-Anilino-1-Naphthalene Sulfonate (ANS) were acquired (Beijing, China). 

### 2.2. Phosphorylation Treatment of SPI

#### 2.2.1. Treatment of Proteins with Sodium Hexametaphosphate

An amount of 10% (*w*/*v*) defatted soy flake solution was adjusted to pH 8.0 and stirred for 2 h to extract protein and then centrifuged (13500× *g*, 30 min) at 4 °C. The supernatant was adjusted to pH 4.5 with 2 mol/L HCl and centrifuged (3300× *g*, 20 min). The pellet was washed twice with distilled water and put into neutralization and modification tank. Sodium hexametaphosphate (SHMP) was added to the neutralization and modification tank so that the percentage of SHMP mass in the solids mass of soy protein solution was 0.4, 0.8, 1.2, 1.6, and 2.0%, respectively. The samples were spray-dried after mixing well to obtain SHMP phosphorylated modified SPI samples, noted as SHMP-0.4%, SHMP -0.8%, SHMP-1.2%, SHMP-1.6%, and SHMP-2.0%, respectively, while the sample not including the adding of sodium hexametaphosphate (SHMP) was utilized as the control one and recorded as SPI.

#### 2.2.2. Treatment of Proteins with Sodium Tripolyphosphate

An amount of 10% (*w*/*v*) defatted soy flake solution was adjusted to pH 8.0 and stirred for 2 h to extract protein and then centrifuged (13500× *g*, 30 min) at 4 °C. The supernatant was adjusted to pH 4.5 with 2 mol/L HCl and centrifuged (3300× *g*, 20 min). The pellet was washed twice with distilled water and put into neutralization and modification tank. Sodium tripolyphosphate (STP) was added to the neutralization and modification tank so that the mass of sodium tripolyphosphate (STP) as a percentage of the mass of solids in soy protein sol was 0.5, 1.0, 1.5, 2.0, and 2.5%, mixed well and spray-dried to obtain STP phosphorylated modified SPI samples, recorded as STP-0.5%, STP-1.0%, STP-1.5%, STP-2.0%, and STP-2.5%, respectively, while the sample without the addition of sodium tripolyphosphate (STP) was used as the control one and recorded as SPI.

### 2.3. Determination of the Level of Phosphorylation

The phosphorylation degree was measured using the protocol of Ai and Jiang [16] with minor adjustments. The phosphorylation level was conveyed as g of phosphorus per g of protein, using the molybdenum blue colorimetric assay to estimate the phosphorus quantity of the samples and employing unmodified SPI as a blank.

### 2.4. Particle Size and Turbidity Circulation 

The protein samples were suspended in H_2_O to prepare a 0.01 g mL^−1^ mix and then added to the measurement cell. The refractive index of the particles was decided at 1.460, where it was adjusted for the dispersant at 1.330, and the particle size distribution characteristics and protein dispersion index (PDI) were estimated via a nanoparticle size and possibility analyzer.

### 2.5. Zeta Potential and Surface Hydrophobicity (H0)

The protein samples were suspended in H_2_O to prepare a 0.01 g mL^−1^ mix and the ζ-potential of each sample was determined via a nanoparticle size and capacity analyzer at a cuvette length of 1 cm and a spacing of 0.4 cm. The hydrophobicity of the protein surface was estimated by reference to the assay of Ma et al. [17] with minor adjustments. Different samples were dispersed in dH_2_O and then these sample mixes were thinned with dH_2_O to a range of concentrations of 0.02, 0.04, 0.06, 0.08, and 0.10 mg mL^−1^. A 5 mL amount of different concentrations was taken and 20 μL of 8-anilinonaphthalene-1-ate (ANS) mix of 8 mmol L^−1^ was added. The samples were mixed thoroughly and placed under light-proof conditions for 15 min. The fluorescence strength of the samples was estimated via a fluorescence spectrophotometer. The concentration of protein is the horizontal axis, the relative fluorescence intensity is the horizontal axis, and the slope of the fitted line is the hydrophobicity mark (H0).

### 2.6. Free Sulfhydryl (SH) and Disulfide (SS) Bonds 

The determination of protein free sulfhydryl groups was based on the approach of Yan et al. [18] with minimal adjustments. The free sulfhydryl quantity of the protein samples was determined using the DTNB (5,5’-dithiobis-(2-nitrobenzoic acid)) method. The protein sample was first prepared in dH_2_O to 0.01 g mL^−1^, then 40 μL of the protein mix was blended with 160 μL of Tris-Gly Urea buffer, mixed well, and then 8 μL of DTNB reagent was inserted and the reaction was run for 20 min at room temperature (RT). The free sulfhydryl concentration was determined as:

$${SH}_{F}=\frac{73.53\times D\times A_{412}}{C}$$

where *SH_F_*—molar concentration of free sulfhydryl mass, μmol g^−1^; 73.53—molar extinction coefficient of DTNB reagent; D—dilution multiple; $A_{412}$—absorbance value of the sample; C—protein dose, mg mL^−1^.

A 1.0 mL sample of 0.01 g mL^−1^ protein mix was inserted to 10.0 mL of Tris-Gly Urea buffer followed by 0.2 mL of β-mercaptoethanol and then responded at 25 °C for 1 h. At the edge of the reaction, 100 mL of 12% trichloroacetic acid (TCA) was added for 1 h, followed by centrifugation at 4000 rpm for 10 min. The supernatant was then showered again with 100 mL of 12% TCA and centrifuged three times. After dissolving the precipitate with 10 mL Tris-Gly Urea buffer and adding 0.1 mL of 4 mg mL^−1^ DTNB, it was mixed well and reacted for 20 min at 25 °C. After the reaction, the absorbance value at 412 nm was quickly estimated and the total sulfhydryl quantity and disulfide bond content were calculated as:

$${SH}_{T}=\;\frac{73.53\times A_{412}}{C}$$

$$\mathrm{SS}=\frac{SH_{T}-SH_{F}}{2}$$

where *SH_T_*—mass molar dose of total sulfhydryl clusters, μmol g^−1^; SS—mass molar dose of disulfide bonds, μmol g^−1^; 73.53—molar extinction coefficient of DTNB reagent; $A_{412}$—absorbance value of the sample; C—protein dose, mg mL^−1^.

### 2.7. Solubility 

The solubility of proteins was estimated by reference to the approach of Kornet et al. [19]. The protein was configured with dH_2_O to a protein solution of 0.01 g mL^−1^. The protein mix was centrifugated at 1000× *g* for 30 min at RT. The amount of protein contained in the upper phase was measured for M1 and a standard curve was made via bovine serum protein as the ideal protein for M2. Protein solubility was then expressed as:

Solubility = M1/M2 × 100%

### 2.8. Size Exclusion Chromatography (SEC)

The experimental approach of Liu et al. [20] was modified for the purpose of determining the molecular weight of the proteins. The protein samples were processed to 0.01 g mL^−1^ and dissolved in dH_2_O. High-performance liquid chromatography (HPLC) was utilized to analyze the molecular weight distribution. An Advanced Bio SEC column was used at a temperature of 25 °C, with dH_2_O as the mobile phase, a flow rate of 0.8 mL min^−1^, a sample injection volume of 20 μL, and a detection wavelength of 280 nm. At 280 nm, the detecting wavelength was set.

### 2.9. Sodium Dodecyl Sulfate–Polyacrylamide Gel Electrophoresis (SDS-PAGE)

The protein was dispersed in dH_2_O to form a 0.01 g mL^−1^ protein solution by the method of Ma et al. [21]. The protein blend (200 μL) was blended with 50 μL of “5 × protein loading buffer solution” and the sample was simmered for 5 min. The sample volume was 5 μL for marker and 10 μL for sample. The gel was first run at 50 V for 30 min, then at 80 V for a further 30 min, and finally at 120 V to stop electrophoresis when the sample turned to the bottom side of the gel. After electrophoresis stopped, the gel was removed and the gel was stained with G-250 stain for 30 min, after which the stain was rinsed off with dH_2_O until the background became apparent. The gels were snapped with a gel imager.

### 2.10. Fourier Transform Infrared (FTIR)

With a few minor adjustments to Peng’s experimental procedure [13], Fourier infrared spectroscopy was used to identify the secondary structure of the proteins. A 100 mg sample of KBr and a 1 mg protein sample were properly combined before being pressed. The absorption spectra were obtained with a shift of 64 scans and a resolution of 4 cm^−1^ over the wavelength range of 4000–400 cm^−1^. Peakfit 4.2.0 software was used to calculate the relative content of each secondary structure by using the integral area. Of these, the band from 1646 to 1662 cm^−1^ could be assigned to the α-helix. The bands from 1608 to 1622 cm^−1^ could be assigned to the anti-parallel intermolecular β-sheet (β1), and the bands from 1622 to 1637 cm^−1^, as well as bands from 1682 to 1700 cm^−1^, could be assigned to the intramolecular β-sheet and parallel intermolecular β-sheet (β2) of the protein, respectively. The band from 1637 to 1645 cm^−1^ could be assigned to the random coil structure, while the bands from 1662 to 1681 cm^−1^ could be assigned to the β-turn.

### 2.11. Intrinsic Fluorescence

The approach of Zhao et al. [22] was used to determine the intrinsic fluorescence of proteins, with a few minor adjustments. A 0.1% protein solution was created by dissolving the protein sample in dH_2_O. The excitation and emission slits were both set at 5 nm, the scanning speed was 12,000 nm min^−1^, and the excitation wavelength was 290 nm. The scanning divergence wavelength ranged from 300 to 400 nm.

### 2.12. Transmission Electron Microscope (TEM)

The microstructure of the protein was examined via transmission electron microscopy. The proteins were dispersed in distillation to form a 0.01 g mL^−1^ protein mix, then an appropriate amount of protein mix was added dropwise to the copper netting, and an appropriate amount of 1% phosphotungstic acid solution (ready-to-use) was added dropwise to stain the copper mesh for 60 s before the protein solution dried completely. After drying, the microstructure of the protein was later examined by TEM with a hastening voltage of 80 kV.

### 2.13. Characteristics of Emulsion

#### 2.13.1. Preparation of Emulsion

To create a protein mix with a concentration of 0.01 g mL^−1^, the protein sample was dispersed in H_2_O. To create an emulsion, a 3:1 mixture of the protein solution and soybean oil was dispersed at a high rapidity of 12,000 r min^−1^ for 1 min.

#### 2.13.2. Emulsifying Activity Index (EAI) and Emulsifying Steadiness Index (ESI) of Emulsion

The emulsion emulsification activity and emulsion stability were estimated with slim adjustments following the protocol of Yan et al. [23]. A 7 mL amount of soybean oil and 21 mL of 0.01 g mL^−1^ protein solution were mixed and dispersed at high speed for 1 min at 12,000 r min^−1^, so 50 μL of the emulsion was combined to 5 mL of 0.1% sodium dodecyl sulfate (SDS) mix from the bottom of the blend at 0 and 10 min, respectively. The absorbance was recorded at 500 nm to obtain the emulsification action index (EAI) and emulsion solidity indicator (ESI). The EAI and ESI are calculated as:

$$\mathrm{EAI}=\;\frac{2\times 2.303\times \mathrm{DF}\times A_{0}}{\left(1-\theta \right)\times c\times L\times 10000}$$

$$\mathrm{ESI}=\;\frac{10\times A_{0}}{\;A_{0}-A_{10}}$$

where DF—100, dilution multiple; *θ*—0.25, volume fraction of oil phase; c—protein concentration, g mL^−1^; L—cuvette light range with 0.001 m.

#### 2.13.3. Morphology Examination of Emulsion

Emulsion globules were examined via confocal laser scanning microscopy (CLSM). The product was homogenized 3 times at high pressure using a high-pressure homogenizer at 80 Mpa. Nile blue and Nile red were prepared into 1% and 0.1% staining solution, respectively, using isopropyl alcohol, and sifted via a 0.22 μm purify tissue to remove the residue. The emulsion was diluted 5 times and 1 mL was taken, then 45 and 40 μL of Nile blue and red were added, respectively, and agitated for 30 s and stained for 30 min, protected from light. At the end of staining, 3 μL of the emulsion was positioned on a slip.

#### 2.13.4. Interfacial Protein Intensity (Γ) of Emulsion

A 5 mL amount of the emulsion was centrifuged at 10,000× *g* for 1 h in a 10 mL centrifuge tube. After centrifugation, the subnatant was eliminated by needle and sifted via a 0.45 μL layer and collected. The protein content of the original emulsion and the remainder was estimated using a BCA kit. The interfacial protein content was calculated as:

$$\Gamma =\frac{W_{T}-W_{S}}{W_{T}}\;$$

where $W_{T}$—protein quantity of the emulsion, mg; $W_{S}$—mass quantity of the protein in the filtrate, mg.

#### 2.13.5. Interfacial Tension of Emulsion

Various materials’ surface tension was estimated via an automated surface tensiometer (DCAT21, Data Physics Instruments GmbH, Stuttgart, Germany) [14]. An amount of 20 mL of the sample mix was then put into a 25 mL cylinder after the protein sample had first been dissolved in dH_2_O (1%, m/v). The apparatus’s measuring variety was always between 1 and 100 mN m^−1^, with an SD that never went beyond 0.03 mN m^−1^.

#### 2.13.6. Rheological Measurement

The determination of the rheology of the emulsions was based on the experimental method of Sun and Arntfield [24] with slight adjustments. High-pressure homogenized emulsions were positioned between two analogous plates of 40 and 1 mm spacing, and frequency scanning tests were accomplished in the assortment of shear frequency 0.1–10 Hz to analyze the variation of elastic modulus (G′) and viscous modulus (G″) of the emulsion samples as well as shear frequency.

### 2.14. Statistical Analysis

All investigations in this study were repeated 3 times and all investigational statistics were portrayed as mean ± SD. The data were analyzed for significance of differences using SPSS ver., 22.0 software, Origin 2021 software for graphing, and *p* < 0.05 was the significance test.

## 3. Results

### 3.1. Degree of Phosphorylation

**Figure 1:**
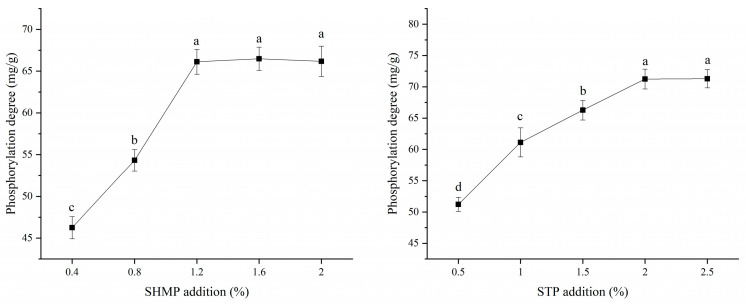
Effect of phosphate addition on phosphorylation level of soybean protein isolate. Note: Values with a different letter(s) indicate a significant difference at *p* ≤ 0.05.

One of the control markers for detecting phosphate on modified soy protein during industrialization is the degree of phosphorylation. As seen in Figure 1, the degree of phosphorylation of SPI followed a trend of initially rising and subsequently decreasing with the addition of phosphate. When they were 1.2% and 2.0%, respectively, the addition of SHMP and STP had achieved its limit. The STP group had a greater level of phosphorylation than the SHMP group. The possibility is that more phosphate groups bind to protein because of the increased likelihood of collision between phosphate groups and the amino acids serine, threonine, and tyrosine in soybean protein at higher phosphate concentrations. However, if the concentration of phosphate is too high, the phosphate groups attached to the altered protein molecules will increase the electrostatic repulsion between protein molecules, leading to a spatial effect that decreases the likelihood of binding phosphate groups to soy protein molecules, which is unfavorable to the phosphorylation process [25]. The SHMP-1.2% group has a lower level of phosphorylation than the STP-2.0% group, which suggests that cyclic metaphosphate and protein react with less efficiency than linear STP [26]. Cyclic metaphosphates, on the other hand, are said to be more effective in chemical reactions [27] (compared to straight chain polyphosphates). Our experimental findings were consistent with earlier ones. According to research by Hu et al. [28], the structural characteristics and composition of proteins may also affect how protein molecules and phosphate groups interact. The environment during protein modification affects the free radical activity in the SPI molecule, and the microenvironments provided by SHMP and STP vary during the phosphorylation of soy proteins. Therefore, the phosphorylation in the STP group is greater than that in the SHMP group because the affinity between SPI and STP is larger than that of SHMP. 

### 3.2. Particle Size Distribution and Turbidity Analysis

**Figure 2:**
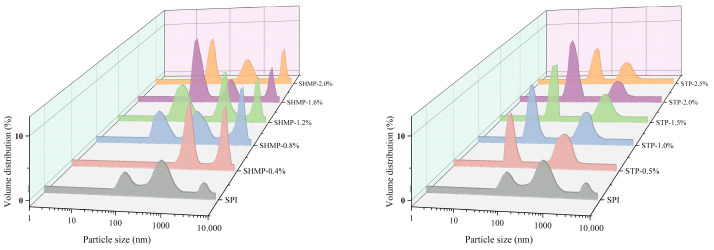
Effect of phosphate addition on particle size distribution of soybean protein isolate.

The impacts of various doses of SHMP (0, 0.4, 0.8, 1.2, and 1.6%) and STP (0.5, 1.0, 1.5, and 2.0%) treatments on the mean particle size and turbidity of the SPI samples are portrayed in Figure 2 and Table 1. The mean particle size and turbidity of SHMP-SPI were larger than those of the control, while the opposite was true for STP-SPI. Triplet distribution curves of approximately 90, 800, and 2100 nm were observed in the SHMP-SPI. The STP samples showed a bimodal distribution curve. The research conducted by Sung et al. [29] showed that the phosphate group of phosphate can selectively induce the reaction to change the protein structure by binding to –OH and –NH_2_ on the edge chain of the protein. Thus, in the industrial spray-drying of modified soy protein, the selective induction of the phosphate group of SHMP with hydroxyl groups and -NH_2_ clusters on the adjacent chain of SPI leads to a partial unfolding of the SPI molecular structure, which promotes the cross-linking of SPI and SPI in the subsequent process because of its ring structure. This leads to an expansion in the size of all SHMP-SPI compared to the unmodified treated SPI particles. As the SHMP dose raises from 0.4% to 1.2%, the average particle size of SHMP-SPI starts to show a decreasing trend. The increase in SHMP concentration increases the number of charged groups on the surface of SPI molecules by cross-linking more phosphate groups, which leads to the formation of more electrostatic repulsions around the SPI molecules. This inhibits the intermolecular interaction forces of SHMP-SPI and increases the spatial potential resistance effect between protein particles [30], causing a decrease in particle size and turbidity. However, as the SHMP dose expanded from 1.2 to 2.0%, the average particle size and turbidity of SHMP-SPI started to show an increasing trend. Xiong et al. [31] demonstrated that an extra phosphate dose can cause the creation of new and large protein accumulates by the enhancement of the binding effect of protein molecules during phosphorylation. Therefore, when the addition amount of SHMP is too high, the electrostatic repulsion between SPI molecules initiated by the addition of SHMP cannot eventually overwhelm the attraction (Van der Waals force and hydrophobicity) and an aggregation phenomenon will take place, resulting in the increase in SHMP-SPI particle size, turbidity, and uneven distribution. While STP is a chain-like phosphate, SHMP has stronger affinity and higher phosphorylation compared with SPI. STP can also augment the electrostatic revulsion and steric hindrance impact of SPI molecule surface by changing the non-covalent interaction within the molecule, resulting in the lessening of particle size and turbidity of STP-SPI. Moreover, too high an STP concentration will also lead to the enhancement of SPI molecular interaction, developing in the increase in particle size and turbidity. However, it is worth noting that the particle size of STP-SPI is always lower than that of SPI, while that of SHMP-SPI is much higher. This indicates that SHMP phosphorylation promotes SPI aggregation to form large-size SPI, while STP phosphorylation results in SPI disaggregation to form small particles with more uniform distribution. The reason may be that the modification of SPI by phosphate is obviously related to the kinds, spatial structure, and molecular properties of phosphate, and SHMP has a ring structure, and the chain length is longer than STP with a chain structure. Consequently, it might be assumed that the impact of SHMP was lower to that of STP due to the longer chain length. Jin et al. [32] reached the same conclusion by studying the impact of phosphate on the gelling features of heat-induced egg white gel. 

### 3.3. SDS–Polyacrylamide Gel Electrophoresis

**Figure 3:**
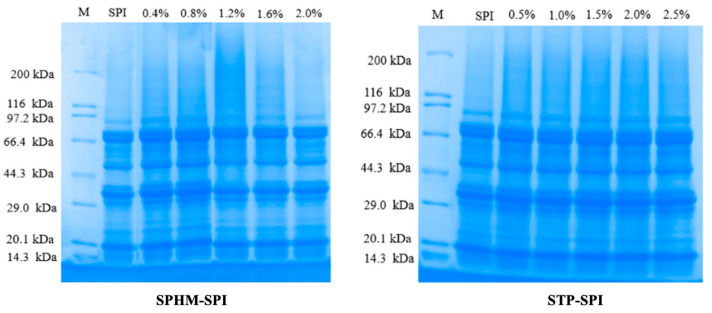
Effect of phosphate addition on gel electrophoresis of soybean protein isolate.

The reduction agent (β-mercaptoethanol) disrupts the hydrophobic interactions between the disulfide bond and the SPI subunits, leading to subunit dissociation, so that SDS-PAGE can essentially signify the differences in the creation of the SPI subunits by phosphate [17]. As can be seen in Figure 3, the overall protein’s structure was not altered by the phosphorylation modification, and the disappearance of the A subunit (SHMP-1.2% group) was more likely due to the cross-linking of non-disulfide covalent bonds [4]; phosphorylation treatment changed one mutual state between subunits and substrates, but had no significant effect on subunits [33], and the remainder of the groups showed insignificant variations between bands of the same molecular weight, implying that the peptide groups in the SPI were not damaged by the introduction of phosphate and that they still maintained their primary structural integrity [26]. Blended with the findings of particle size and turbidity tests, the phosphorylation treatment promoted the aggregation or depolymerization of SPI molecules from SPI molecules, indicating that phosphate can significantly affect the stability of proteins in aqueous solution.

### 3.4. Fourier Transform Infrared 

**Figure 4:**
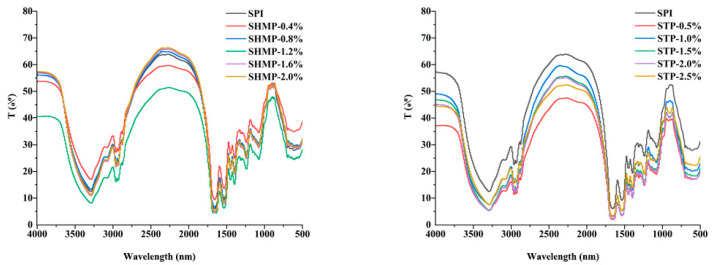
Effect of phosphate addition on FTIR spectra of soybean protein isolate.

Both STP and SHMP exhibit comparable properties in their infrared spectra. As seen in Figure 4, SPI exhibits three key distinguishing peaks: the amide I band (mostly C=O stretching) at 1480–1575 cm^−1^; the amide II band (C–N stretching combined with N–H bending) at 1600–1700 cm^−1^; and the amide A band (related with N–H stretching coupled with H–bonding) at 3273 cm^−1^ [34]. Protein structural changes are indicated by variations in spectral peak location and intensity [35]. The stretching vibration of P-O in PM-SPI samples, in contrast, was primarily responsible for the STP-SPI’s unique absorption peak at 895 cm^−1^ and rising intensity with increasing STP concentration. Electrostatic interactions between proteins and other components were linked to changes in amide I and II [36].

Table 2 displays the secondary structures of the STP and SHMP alterations. After phosphorylation, the α-helical content of SPI decreased while the β-fold and irregular curl content increased. As phosphate addition increased, the α-helical content of SPI displayed a trend of first increasing and then decreasing while the irregular curl content displayed a trend of first increasing and then decreasing. Its irregular curl content peaked when SHMP and STP were added at 1.2% and 2.0%, respectively, and the STP-2.0% group was higher than the STP. Protein aggregation and the side chains of amino acids may have changed, which is why the secondary structure of SPI changed before and after alteration [37]. Amide-I is characteristic of the coiled-coil composition of proteins, and the overview of phosphate may affect the α-helical coiled-coil structure of proteins primarily by increasing the repulsive forces between charged residues in the protein chain [38]. Phosphorylation of STP also stemmed in a decline in the amides I and II band peak amplitude. These alterations suggest that these reactive groups are reduced by binding to the phosphate group. Changes in the side chain rearrangement or secondary structure of peptides in proteins result in changes in peak intensity and peak position. The phosphorylation reaction acts on only a few particular amino acid remains, not most of them. Both the ε-NH_2_ of Lys residues and the hydroxyl of Ser or Thr remains show the greatest effects [39]. It can be speculated that a C-N-P or C-O-P bond may have been established between SPI and STP or SHMP. This is the same result as that reported by Zhao et al. [26].

### 3.5. Intrinsic Fluorescence Emission Spectra

**Figure 5:**
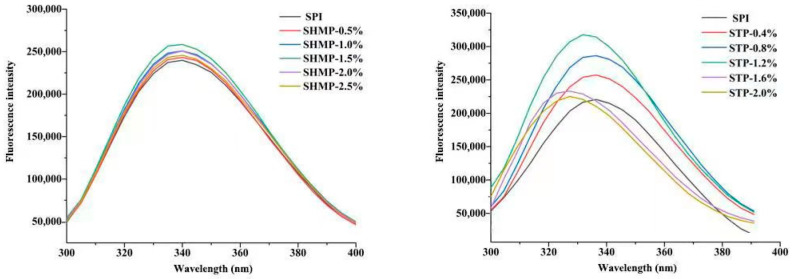
Effect of phosphate addition on intrinsic fluorescence emission spectra of soybean protein isolate.

When the milieu changes, inherent fluorescence might react, and the phosphate-based tertiary structure of SPI Try persists. According to the findings in Figure 5, there was a tendency for the fluorescence intensity of SHMP-SPI and STP-SPI to increase and subsequently decrease when the phosphate concentration increased. Although there was a minor blue shift, the fluorescence intensity of SHMP-SPI did not significantly alter with SHMP concentration. However, the total fluorescence intensity of STP-SPI was higher than that of SHMP-SPI, and the fluorescence lightness of STP-SPI showed a considerable change with increasing STP concentration as well as a substantial blue shift. A strong negative association between the amount of α-helices and the surface hydrophobicity was found by Kato et al. [40]. A decrease in structural ordering, as shown by a decrease in the α-helical structure of the secondary structure, is caused by phosphorylation treatment, which also causes an increase in the hydrophobic region within the SPI molecule. Exposure of the hydrophobic sites within the molecule may also cause the aromatic amino acid residues in the soy protein aggregates to move from a hydrophilic to a hydrophobic environment [41], leading to the blue-shift phenomenon. The fluorescence strength and supreme excitation wavelength (λmax = 340.7) of the SHMP-treated protein did not alter more than that of the STP-treated protein (λmax = 335.6); this is due to the combination of different modes of aggregation occurring between SPI molecules and the unfolding of the SPI molecular structure after phosphate treatment reduces the internal burst, and SPI molecules are more likely to form large aggregates and partial expansion of the molecules during SHMP treatment, reducing the acquaintance of the protein interior color amino acid remains, so the fluorescence intensity changes are not as pronounced as for STP-SPI. When the phosphate concentration continues to increase, the aggregation behavior leads to more tryptophan residues buried, and a too-high concentration of phosphate leads to SPI re-aggregation and increased spatial site resistance, resulting in fluorescence burst and reduced fluorescence strength [20]. The results of endogenous fluorescence suggest that SHMP and STP modification can cause changes in the conformation of SPI tertiary structure.

### 3.6. Surface Hydrophobicity and ζ-Potential

The surface hydrophobicity (H0) and zeta potential of proteins play a crucial part in determining the structural conformation of phosphorylated modified SPI by judging the changes in intermolecular interaction forces and can influence the functional properties of modified SPI [42]. The surface hydrophobicity and ζ-potential of the two phosphate-modified soybean proteins is shown in Table 1. Both SHMP and STP treatments stemmed in an expansion and then a decline in the surface hydrophobicity and ζ-potential of SPI. Compared to SPI, the H0 of SPI reached the maximum at SHMP-1.2% and STP-2.0% and the ζ-potential of SPI was highest at SHMP-1.6% and STP-2.0%. It is hypothesized here that the disclosure of hydrophobic groupings and the expansion in surface charge number are caused by alterations in the secondary and tertiary structure of SPI. The addition of phosphate leads to the denaturation/unfolding of the SPI molecule subjecting the hydrophobic sites and hydrophobic and charged groups. Moreover, phosphate colonization also causes a growth in the negative charge groups on the protein exterior [43], which leads to a corresponding rise in hydrophobicity and ζ-potential on the phosphate-modified SPI surface. However, overly high concentrations of phosphate may alter the interaction between protein side chain groups and phosphate through inter-molecular aggregation and swelling of SPI and intra-molecular hydrophobic association aggregation of SPI, inhibiting additional disclosure of hydrophobic and charged parties, and as a substitute reburying them within the protein molecule through aggregation [44], reducing the surface hydrophobicity and ζ-potential values. However, the extent of H0 and ζ-potential changes were more pronounced in STP-SPI compared to SHMP-SPI. This may be due to the increase in the disordered structure of SPI during STP phosphorylation treatment, which improves the flexibility of SPI molecules by forming a more extended and sparser structure [45]. The above results together contribute to make some hydrophilic groups of STP-SPI such as -COO- and -OH and hydrophobic regions will be exposed to a higher degree, leading to a more pronounced degree of H0 and ζ- potential changes. The STP-SPI particles with higher zeta potential have increased electrostatic repulsion between ions and the system is relatively stable because their charges inhibit the aggregation of SPI and enhance their stability [46]. This causes the protein molecules in STP-SPI to show small particle dispersion in the liquid that is reliable with the particle size findings.

### 3.7. Free Sulfhydryl Groups and Disulfide Bonds

The sulfhydryl disulfide exchange (SH/SS) reaction is an important factor in altering the spatial structure of industrially phosphorylated modified SPI and plays an essential function in intermolecular accumulation and depolymerization of SPI [47]. The results for free sulfhydryl groupings and di-sulfide bonds for SHMP-SPI and STP-SPI are shown in Table 1. After the industrial phosphorylation modification, both the free sulfhydryl groups of SPI revealed a tendency of rising and then declining, and the trend in disulfide bonds revealed a tendency of declining and then expanding. As the concentration of SHMP increased from 0.4 to 1.2%, the free sulfhydryl content increased from 2.91 to 14.79 umol g^−1^, whereas the free sulfhydryl quantity of STP-SPI did not increase drastically. In contrast, the disulfide bond content of STP-SPI decreased more than that of SHMP-SPI. Phosphorylation appears to lead to exposure of the -SH group in the SPI and disruption of the disulfide bond due to the negative charge introduced by phosphorylation and the subsequent shift in the unfolding structure of the SPI [48]. The accumulation of phosphate leads to a relaxing impact on the SPI structure by increasing the negative charge on the SPI exterior, exposing the buried sulfhydryl groups in the protein in the process of reducing the size of the SPI particle, leading to an expansion in cross-linking of free sulfhydryl groups and the breaking of disulfide bonds, the breaking of which stabilizes the SPI accumulates, leading to an expansion in the rearrangement and hydrophobicity of the SPI particles. However, the change in -SH content was more pronounced in the SHMP-SPI group, which may be due to the structural unfolding of soy protein, causing the conversion of other sulfur-containing compounds into free sulfhydryl groups in addition to disulfide bonds [49]. The transformation of free sulfhydryl groupings into disulfide bonds resulted in the burial of some free -SH groups by hydrophobic interactions and the phosphorylation of some free -SH groups to form intra- or intermolecular -SS. This agrees with the particle size and hydrophobicity results. The results suggest that phosphorylation can regulate the polymerization and depolymerization between SPI molecules that in turn influences the functional features of the protein [12], and similar conclusions were reached by the study.

### 3.8. Transmission Electron Microscope

**Figure 6:**
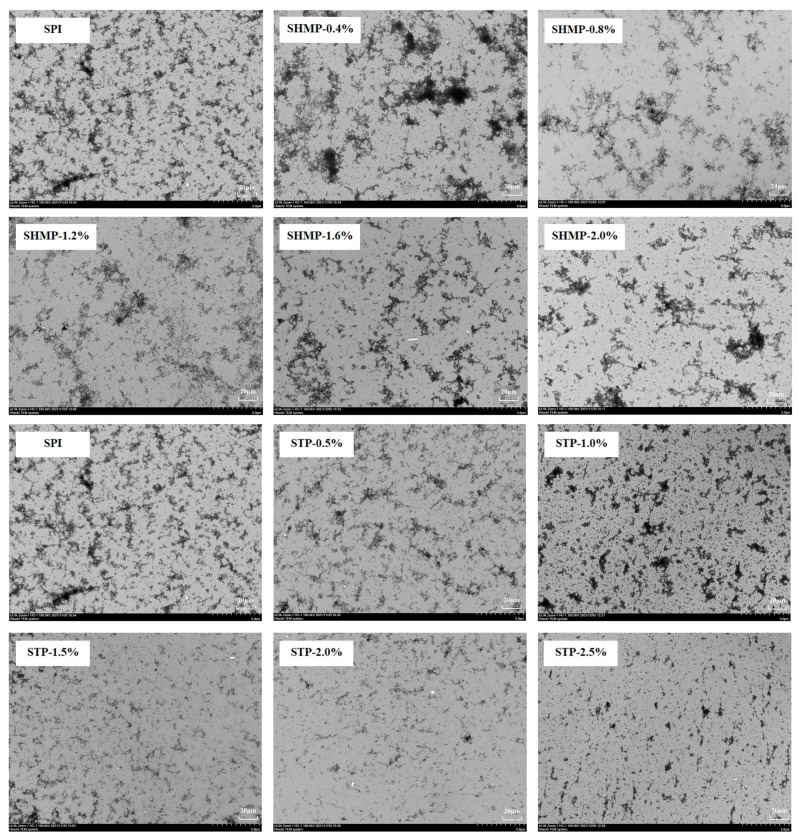
Effect of phosphate addition on backbone structure of soybean protein isolate.

Figure 6 shows the TEM snaps of SHMP-SPI and STP-SPI. The backbone morphology of the protein was examined by TEM. As can be noted from the state portrayed in Figure 6-SPI, the native SPI is in small, aggregated bundles and after industrial phosphorylation treatment, the SHMP-0.4% group shows strong black centers of aggregation, and the stacked SHMP-SPI shows a high skeletal rigidity forming a multi-branched structure. With increasing concentration of SHMP, the SPI aggregates showed depolymerization and repolymerization, while STP-SPI also showed aggregation–depolymerization–repolymerization, but STP-SPI showed smaller aggregates and did not show a large aggregation structure like that of SHMP-SPI, the white particles being the SPI particles suspended in solution. During industrial phosphorylation, phosphate and protein interact primarily through hydrophobic and electrostatic interactions, with phosphate acting as a bridge to polymerize protein molecules [50]. 

### 3.9. Solubility

The solubility of SHMP-SPI and STP-SPI is shown in Table 3. The phosphorylated SPI is more soluble than the unphosphorylated SPI. The STP-SPI group showed a greater improvement in solubility than the SHMP-SPI group, with the soybean isolate having the maximum solubility (97.09%) in the STP-2.0% treatment group and the SPI having the lowest solubility (93.64%) in the SHMP-1.2% treatment group. The SPI side chain groups undergo extensive anionic phosphate group introduction during industrial phosphorylation, and the phosphate groups connected to the SPI molecules are able to make many hydrogen bonds with water molecules [30]. The electrostatic repulsion between the SPIs in aqueous solution is increased by increasing the negative charge introduced by the phosphate group along the surface of the SPIs [26]. High phosphate concentrations limit the interaction between the inhibited protein and H_2_O molecules, causing phosphate and SPI molecules to compete with H_2_O molecules and reducing SPI solubility [12]. The STP-2.0% group had the highest solubility, which could be attributed to the linear structure of STP, the formation of more H-ion ligands in the STP phosphate group during industrial phosphorylation, the presence of more positive and negative charges on the surface of the protein molecules, an increase in the number of H-bonding interactions between SPI molecules, the thickness of the hydration layer, and the ability of hydrophilic and hydration repulsion to repel one another [51].

### 3.10. Emulsifying Action Index and Emulsion Solidity Index 

The EAI and ESI of STP and SHMP at different concentrations are shown in Table 3. With increasing phosphate addition, the emulsifying action (EAI) and emulsion solidity (ESI) of SPI revealed a tendency of first rising and then declining; the highest rates were reached when SHMP and STP were added at 1.2% and 2.0%, respectively, and the STP-2.0% group was better than the SHMP-1.2% group. Moderate phosphorylation led to unfolding of the protein secondary structure, displaying more phosphorylation sites and hydrophobic groupings, and increasing the intermolecular flexibility of the SPI [11]. During emulsion preparation, the high-pressure homogeneous sheer force of the SPI facilitated diffusion to the interface and increased the interfacial protein content, and the phosphorylated SPI facilitated protein adsorption to the oil–water interface and oil droplet dispersion [10]. Alternatively, the improved water solubility of SPI also has an impact on improved emulsification, as the SPI molecular aggregates tend to adhere to the oil–water interface and form a thicker adsorption layer, changing the amphiphilic nature of the SPI attached to the oil–water interface, resulting in better emulsifying activity [52]. The adding of phosphate leads to increased irregular curl content and increased structural flexibility of the SPI structure, which reduces the surface tension of the emulsion and makes it simpler to create emulsion droplets, while increasing the electrostatic repulsion between droplets, making it easier for droplets to disperse, thus significantly improving emulsion stability [53]. This is a good illustration of how phosphorylation can quicken the rise in electrostatic repulsion, thus promoting the dispersion of emulsion droplets and inhibiting the incorporation of oil droplets. Smaller particle sizes and higher ζ-potential values have been reported to increase electrostatic repulsion and spatial site resistance among droplets, and phosphorylation-promoted adsorption layers lead to increased electrostatic repulsion at interfacial proteins, preventing aggregation and flocculation of emulsion particles [54]. Emulsion stability is one of the most improved properties, as confirmed by the data in Table 3.

### 3.11. Confocal Laser Scanning Microscopy 

**Figure 7:**
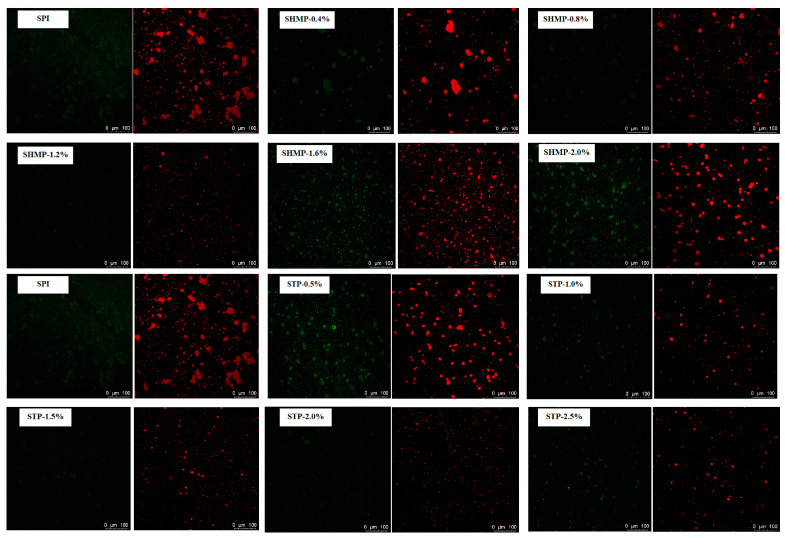
Effect of phosphate addition on microstructure of emulsion prepared by soybean protein isolate.

The microstructure of emulsion particles has been frequently displayed using a confocal laser scanning microscope, which can immediately display the particle size and dispersion state [55]. The red fluorescent core and green, fluorescent periphery in the CLSM micrographs indicate the oil and protein fractions, respectively. As shown in Figure 7, the size of the phosphorylated SPI droplets decreased, the emulsion was more uniformly dispersed, and the droplet shape tended to be regular spherical compared to the emulsion formed by the non-phosphate modified SPI. With increasing phosphate addition, the droplet dispersion revealed a trend of expanding and then declining. The highest dispersion of the emulsions was observed in the SHMP-1.6% and STP-2.0% groups, and the emulsions were more dispersed in the STP-2.0% group than in the SHMP-1.6% group. The phosphorylation treatment leads to the aggregation of protein molecules, resulting in the rearrangement and relaxation of the interface during emulsification, which fails to form a stable interfacial film, resulting in large areas of red oil droplet aggregation. With the addition of phosphate to increase the surface charge of SPI, the resulting interfacial protein film is able to induce strong enough electrostatic repulsion in adjacent emulsion droplets to offer better spatial solidity and inhibit accumulation and binding between emulsions [28]. On the other hand, the phosphate modification led to the stretching and unfolding of the SPI structure, the exposure of internal lipophilic groups, and the improvement of the flexible structure, which promoted the molecular interaction between the phosphorylated SPI and SPI at the oil–water interface, contributing to the adsorption of SPI at the oil–water interface and the foundation of a stiffer interfacial film, while inhibiting the flocculation of the emulsion droplets [18]. This also explains the minor particle size and improved stability of SPI emulsions after phosphorylation.

### 3.12. Emulsion Rheology 

**Figure 8:**
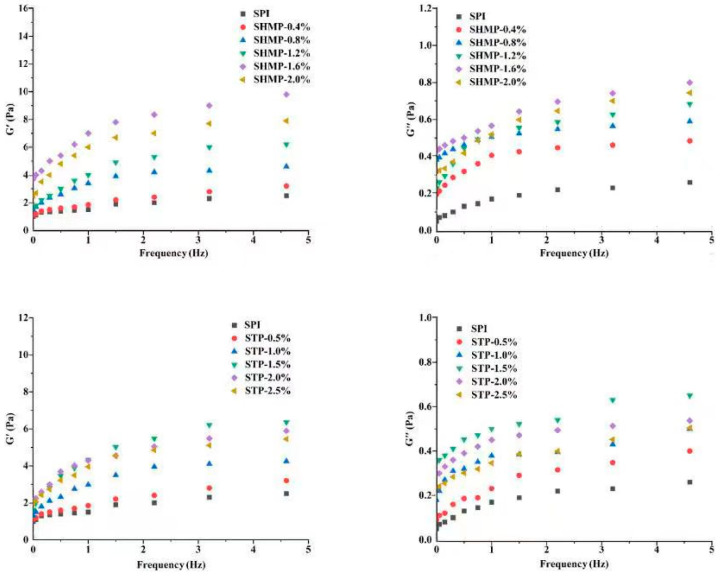
Effect of phosphate addition on rheology properties of emulsion prepared by soybean protein isolate.

By applying different degrees of oscillation frequency strain to the emulsion, elastic modulus G′ is a measurement of elasticity and represents the storage modulus of the energy of stress, which can be restored when the stress is released, while the viscous modulus G″ represents the viscous components which assumes the flow resistance of the sample. G’ and G″ are used to reflect the viscoelasticity of the emulsion, which reflects the structural characteristics and intermolecular interactions of the SPI molecules at the water–oil interface after phosphorylation, which is one of the key factors determining emulsion stability [56]. Figure 8 shows the change in dynamic modulus as the phosphorylated SPI emulsion frequency scanned. The energy storage modulus (G’) and loss modulus (G″) of all emulsion samples expanded with increasing frequency sweep and tended to increase and then decrease with increasing phosphate concentration. The energy storage modulus (G’) and loss modulus (G″) reach their maximum values when SHMP and STP are added at 1.6% and 1.5%, respectively, with G″ values reliably higher than G″ values, implying that the elastic properties dominate and that the emulsion forms a better elastic network structure [57]. The binding among protein molecules via covalent and noncovalent bonds has an important influence on the maintenance of the gel network. Phosphate-modified SPI particles have a higher ζ-potential and higher hydrophobicity (Table 1), which enhances covalent and non-covalent interactions among proteins [58]. The SPI molecular chain is unfolded by phosphorylation [59]; more protein is adsorbed at the oil–water interface and wraps around the oil droplet, increasing the stabilizing effect on the oil droplet and the resulting increase in emulsion viscosity, which increases the energy storage modulus and loss modulus of the emulsion, resulting in improved emulsion stability [21]. At the identical frequency, the G’ values of both SHMP-SPI and STP-SPI were greater than those of the control group, and the G″ values were substantially greater than those of the control group, indicating that the phosphorylated SPI had better viscosity performance. At the same time, the viscosity of the SHMP-modified SPI performed better than that of the STP-modified SPI. The range of G’ dynamics in the SHMP-SPI group was superior to that in the STP-SPI group, while the range of G″ dynamics in the STP-SPI group was superior to that in the SHMP-SPI group, indicating that the larger molecular weight SPI promoted more entanglement in the emulsion [60]. At the same concentration, the higher-molecular-weight SHMP-SPI occupied more space and was more likely to meet other molecules, reducing molecular mobility and increasing viscoelasticity. This explains very well the effect of the two phosphate modifications on SPI.

### 3.13. Interfacial Tension

The interfacial tension of emulsions can characterize the ease of emulsion formation and infer the adsorption and exchange behavior of SPI at the emulsion interface, which is related to the diffusion rate of SPI molecules and their own adsorption capacity. As can be seen from Table 3, the addition of phosphate reduced the interfacial tension and indicated an inclination to decrease and then boost with increasing phosphate addition. The interfacial tension of the STP-2.0% group was shorter than that of the SHMP-1.2% group. On the one hand, phosphates are highly hydrophilic and after industrial phosphorylation, the surface hydrophobicity of SPI molecules increases and the hydrophilic/hydrophobic ratio reaches equilibrium, increasing the rearrangement efficiency of SPI molecules in the interfacial layer after adsorption to the interfacial layer, promoting rapid protein adsorption at the oil–water interface, and reducing the interfacial tension [61]. Alternatively, the integration of the phosphate group increases the net charge on the superficial layer of the SPI molecule and the increased electrostatic repulsion leads to a variation in protein compliance, deconvolution of the ɑ-helix, and a disheveled structure that enables speedy conformational change of the protein at the oil–water interface, exposing the hydrophobic amino acids from the interior structure and, thus, reducing the interfacial tension [14]. It is commonly assumed that the increased β-fold content encourages interactions among proteins at the oil–water interface, adding to the creation of denser and stiffer interfacial films [62], which also leads to a reduction in interfacial tension. Excess phosphate inhibits the dissociation of SPI molecules, reducing the net surface charge, and electrostatic repulsion is insufficient to maintain the stability of the system, leading to aggregation of SPI, encapsulation of hydrophobic groups, and a decrease in surface hydrophobicity. This reduces the rate of SPI adsorption to the interfacial layer and the ability to cross-link with the oil phase and increases the mutual repulsion of the water and oil phases at the contact surface, with a further increase in interfacial tension values. The lower interfacial tension in the STP 2.0% group compared to the SHMP 1.2% group may be due to the smaller molecular size of the proteins in the STP 2.0% group. Proteins with a smaller molecular size and more relaxed conformation have a greater ability to reduce interfacial tension, which is more conducive to rapid protein adsorption at the water/oil interface [63]. The results suggest that the reduction in interfacial tension of the phosphate modified SPI emulsion is one of the key factors contributing to the increase in EAI.

## 4. Conclusions

The structural and functional features of SPI treated with STP and SHMP during industrial production were investigated. It was shown that the absolute values of α-helix content, endogenous fluorescence intensity, surface hydrophobicity, and ζ-potential of SPI in both SHMP and STP groups tended to increase and then decrease with increasing phosphate addition, reaching maximum values at 1.2% and 2.0% for SHMP and STP, respectively. Microscopic results showed that the SHMP-modified SPI exhibited a large and aggregated rigid skeleton structure, while STP exhibited small and black aggregates. The emulsifying activity and emulsion solidity of both SHMP-SPI and STP-SPI demonstrated a tendency of rising and then declining with increasing phosphate adding. The emulsions of SHMP-SPI and STP-SPI have a predominantly elastic behavior and show an increasing trend in viscosity. The phosphorylation of SHMP cross-linked the protein, facilitating the formation of many micelles in solution. These micelles interacted to form a mesh structure and increase the viscosity of the system, making it suitable for use as a thickener in food processing. Phosphorylation of STP reduces the size of protein molecules and increases intermolecular interactions, allowing it to be utilized as a dispersing additive in food processing. This study provides a reference to produce functional properties of industrially modified soy isolate proteins for the food industry.

## Figures and Tables

**Table 1:** Effects of phosphate on average particle size, PDI, turbidity, surface hydrophobicity, ζ-potential, free sulfhydryl groups, and disulfide bonds of soybean protein isolate.

Sample	Concentration	Average Particle Size (nm)	PDI	Turbidity	Surface Hydrophobicity	ζ-Potential (mV)	Free Sulfhydryl Groups (μmol/g)	Disulfide Bonds (μmol/g)
SPI	0	408.82 ± 2.18 ^fA^	0.45 ± 0.02 ^aAB^	0.195 ± 0.007 ^dA^	310.80 ± 6.56 ^eF^	−20.80 ± 0.23 ^eF^	1.2133 ± 0.0125 ^eD^	13.5053 ± 0.1990 ^aA^
SHMP	0.4%	1341.01 ± 7.35 ^a^	0.38 ± 0.05 ^b^	0.252 ± 0.002 ^a^	484.98 ± 7.37 ^d^	−23.34 ± 0.21 ^d^	2.1268 ± 0.1760 ^c^	12.0783 ± 0.4114 ^b^
	0.8%	1140.12 ± 5.56 ^b^	0.31 ± 0.04 ^c^	0.241 ± 0.004 ^b^	945.99 ± 8.00 ^c^	−25.86 ± 0.10 ^c^	2.9100 ± 0.2110 ^d^	10.3210 ± 0.4780 ^c^
	1.2%	567.91 ± 3.56 ^e^	0.18 ± 0.01 ^e^	0.229 ± 0.005 ^c^	1183.00 ± 8.82 ^a^	−29.63 ± 0.18 ^a^	14.7899 ± 0.1210 ^a^	8.6773 ± 0.2212 ^e^
	1.6%	664.47 ± 4.19 ^d^	0.26 ± 0.02 ^d^	0.236 ± 0.001 ^b^	984.00 ± 7.46 ^b^	−29.45 ± 0.12 ^a^	13.2934 ± 0.2930 ^b^	8.6791 ± 0.2103 ^e^
	2.0%	759.64 ± 10.21 ^c^	0.24 ± 0.03 ^d^	0.237 ± 0.002 ^b^	950.00 ± 7.90 ^c^	−28.78 ± 0.13 ^b^	6.0904 ± 0.3010 ^c^	9.1565 ± 0.3980 ^d^
STP	0.5%	299.91 ± 2.12 ^B^	0.36 ± 0.02 ^C^	0.089 ± 0.005 ^B^	1109.10 ± 18.45 ^E^	−25.31 ± 0.23 ^E^	1.3533 ± 0.0124 ^C^	12.1268 ± 0.2212 ^B^
	1.0%	297.73 ± 4.21 ^B^	0.41 ± 0.02 ^B^	0.082 ± 0.001 ^C^	1274.30 ± 16.99 ^C^	−29.82 ± 0.10 ^D^	1.4068 ± 0.0134 ^B^	10.2836 ± 0.1836 ^C^
	1.5%	290.37 ± 3.33 ^C^	0.26 ± 0.02 ^D^	0.080 ± 0.002 ^C^	1479.60 ± 18.31 ^B^	−31.45 ± 0.13 ^C^	1.5059 ± 0.0132 ^A^	9.9836 ± 0.1735 ^D^
	2.0%	266.73 ± 5.63 ^E^	0.11 ± 0.02 ^E^	0.060 ± 0.003 ^D^	1864.50 ± 27.17 ^A^	−35.01 ± 0.12 ^A^	1.4259 ± 0.0101 ^B^	7.5765 ± 0.1134 ^F^
	2.5%	281.53 ± 4.87 ^D^	0.46 ± 0.02 ^A^	0.068 ± 0.006 ^D^	1210.50 ± 22.80 ^D^	−33.68 ± 0.25 ^B^	1.4118 ± 0.0099 ^B^	8.3846 ± 0.1239 ^E^

Note: Comparisons were carried out between values of the same column; values with a different lowercase letter (s) indicate a significant difference at *p* ≤ 0.05 between the sample with SHMP; values with a different capital letter (s) indicate a significant difference at *p* ≤ 0.05 between the sample with STP.

**Table 2:** Effect of phosphate addition on the secondary structures of soybean protein isolate.

Secondary Structure Content (%)		β-Sheet/%	α-Helix/%	β-Turn/%	Random Coil/%
Sample	Concentration				
SPI	0	26.20 ± 0.35 ^eC^	36.18 ± 0.05 ^aA^	15.12 ± 0.03 ^eA^	22.50 ± 0.23 ^eF^
SHMP	0.4%	30.39 ± 0.17 ^d^	25.11 ± 0.01 ^b^	22.50 ± 0.13 ^a^	22.00 ± 0.27 ^e^
	0.8%	31.43 ± 0.12 ^b^	23.74 ± 0.31 ^d^	21.33 ± 0.21 ^c^	23.50 ± 0.12 ^c^
	1.2%	37.34 ± 0.27 ^a^	20.05 ± 0.14 ^e^	3.81 ± 0.32 ^f^	38.80 ± 0.22 ^a^
	1.6%	31.17 ± 0.98 ^c^	23.64 ± 0.03 ^d^	22.19 ± 0.09 ^b^	23.00 ± 0.23 ^d^
	2.0%	31.05 ± 0.54 ^c^	24.55 ± 0.06 ^c^	20.31 ± 0.16 ^d^	24.09 ± 0.03 ^b^
STP	0.5%	26.52 ± 0.84 ^C^	27.24 ± 0.12 ^C^	12.78 ± 0.24 ^C^	33.46 ± 0.01 ^D^
	1.0%	27.05 ± 0.07 ^B^	27.14 ± 0.04 ^CD^	11.66 ± 0.51 ^E^	33.75 ± 0.07 ^C^
	1.5%	27.24 ± 0.15 ^B^	27.04 ± 0.08 ^D^	12.15 ± 0.20 ^D^	34.27 ± 0.12 ^B^
	2.0%	28.39 ± 0.37 ^A^	25.83 ± 0.08 ^E^	10.98 ± 0.35 ^F^	34.80 ± 0.11 ^A^
	2.5%	25.55 ± 0.28 ^D^	27.67 ± 0.02 ^B^	13.22 ± 0.02 ^B^	33.12 ± 0.04 ^E^

Note: Comparisons were carried out between values of the same column; values with a different lowercase letter (s) indicate a significant difference at *p* ≤ 0.05 between the sample with SHMP; values with a different capital letter (s) indicate a significant difference at *p* ≤ 0.05 between the sample with STP.

**Table 3:** Effect of phosphate addition on solubility, emulsifying action, emulsion solidity, and interfacial tension of soybean protein isolate.

Sample	Concentration	Solubility (%)	(EAI) (m^2^/g)	(ESI) (min)	Interfacial Tension
SPI	0	85.86 ± 0.49 ^dE^	25.53 ± 0.123 ^eE^	18.51 ± 0.82 ^eF^	18.470 ± 0.124 ^aA^
SHMP	0.4%	88.44 ± 0.28 ^c^	30.00 ± 0.201 ^d^	35.91 ± 1.90 ^d^	16.406 ± 0.163 ^b^
	0.8%	90.26 ± 0.26 ^b^	33.00 ± 0.167 ^c^	60.89 ± 1.66 ^c^	14.726 ± 0.142 ^cd^
	1.2%	94.64 ± 0.25 ^a^	35.82 ± 0.158 ^a^	87.56 ± 1.00 ^a^	13.340 ± 0.212 ^e^
	1.6%	90.35 ± 0.27 ^b^	35.15 ± 0.103 ^b^	72.80 ± 0.90 ^b^	14.570 ± 0.225 ^d^
	2.0%	86.72 ± 0.19 ^d^	34.65 ± 0.191 ^b^	68.31 ± 0.99 ^b^	15.013 ± 0.109 ^c^
STP	0.5%	89.19 ± 0.26 ^D^	27.00 ± 0.15 ^D^	46.63 ± 1.11 ^E^	15.175 ± 0.205 ^B^
	1.0%	90.68 ± 0.21 ^C^	30.00 ± 0.35 ^C^	70.00 ± 1.38 ^D^	14.246 ± 0.109 ^C^
	1.5%	93.55 ± 0.20 ^B^	35.00 ± 0.29 ^B^	78.35 ± 1.00 ^C^	12.365 ± 0.102 ^D^
	2.0%	97.09 ± 0.21 ^A^	39.50 ± 0.38 ^A^	95.00 ± 1.21 ^A^	11.555 ± 0.163 ^E^
	2.5%	94.53 ± 0.24 ^B^	38.47 ± 0.44 ^A^	90.00 ± 1.09 ^B^	12.887 ± 0.109 ^D^

Note: Comparisons were carried out between values of the same column; values with a different lowercase letter (s) indicate a significant difference at *p* ≤ 0.05 between the sample with SHMP; values with a different capital letter (s) indicate a significant difference at *p* ≤ 0.05 between the sample with STP.

## Data Availability

The data presented in this study are available in [Study on the structure, function, and interface characteristics of soybean protein isolate by industrial phosphorylation].
